# Omicron SARS-CoV-2 mutations stabilize spike up-RBD conformation and lead to a non-RBM-binding monoclonal antibody escape

**DOI:** 10.1038/s41467-022-32665-7

**Published:** 2022-08-24

**Authors:** Zhennan Zhao, Jingya Zhou, Mingxiong Tian, Min Huang, Sheng Liu, Yufeng Xie, Pu Han, Chongzhi Bai, Pengcheng Han, Anqi Zheng, Lutang Fu, Yuanzhu Gao, Qi Peng, Ying Li, Yan Chai, Zengyuan Zhang, Xin Zhao, Hao Song, Jianxun Qi, Qihui Wang, Peiyi Wang, George F. Gao

**Affiliations:** 1 CAS Key Laboratory of Pathogen Microbiology and Immunology, Institute of Microbiology, Chinese Academy of Sciences, Beijing, 100101 China; 2 University of Chinese Academy of Sciences, Beijing, 100049 China; 3 Research Network of Immunity and Health (RNIH), Beijing Institutes of Life Science, Chinese Academy of Sciences, Beijing, 100101 China; 4 College of life Science, Shanxi University, Taiyuan, 030006 China; 5 School of Life Science, University of Science and Technology of China, Hefei, 230026 China; 6 Cryo-EM Center, Department of Biology, Southern University of Science and Technology, Shenzhen, 518055 China; 7 Department of Basic Medical Sciences, School of Medicine, Tsinghua University, Beijing, 100084 China; 8 Central Laboratory, Shanxi Province Hospital of Traditional Chinese Medicine, Taiyuan, 030012 China; 9 Shanxi Academy of Advanced Research and Innovation, Taiyuan, 030032 China; 10 School of Medicine, Zhongda Hospital, Southeast University, Nanjing, 210009 China

**Keywords:** Cryoelectron microscopy, SARS-CoV-2, Viral proteins

## Abstract

Omicron SARS-CoV-2 is rapidly spreading worldwide. To delineate the impact of emerging mutations on spike’s properties, we performed systematic structural analyses on apo Omicron spike and its complexes with human ACE2 or S309 neutralizing antibody (NAb) by cryo-EM. The Omicron spike preferentially adopts the one-RBD-up conformation both before and after ACE2 binding, which is in sharp contrast to the orchestrated conformational changes to create more up-RBDs upon ACE2 binding as observed in the prototype and other four variants of concern (VOCs). Furthermore, we found that S371L, S373P and S375F substitutions enhance the stability of the one-RBD-up conformation to prevent exposing more up-RBDs triggered by ACE2 binding. The increased stability of the one-RBD-up conformation restricts the accessibility of S304 NAb, which targets a cryptic epitope in the closed conformation, thus facilitating the immune evasion by Omicron. These results expand our understanding of Omicron spike’s conformation, receptor binding and antibody evasion mechanism.

## Abstract (web summary)

The SARS-CoV-2 Omicron variant spreads rapidly. Here the authors show that Omicron S preferentially adopts the one-RBD-up conformation, which leads to a non-RBM-binding monoclonal antibody escape. Mutagenesis reveals that S371L, S373P and S375F substitutions enhance the conformational stability.

## Introduction

The coronavirus disease 2019 (COVID-19) pandemic, caused by SARS-CoV-2^1,2^, has posed a major threat to human health and affected the worldwide socio-economy. Continuously emerging variants have brought about remarkable challenges in controlling and preventing SARS-CoV-2 infection. Recently, a new SARS-CoV-2 variant, Omicron, has brought widespread concern. It was first reported from South Africa on November 24, 2021 (also known as lineage B.1.1.529) and was quickly classified as the fifth VOC by the World Health Organization on November 26, 2021, following the Alpha, Beta, Gamma, and Delta variants^3^. By March 2022, it had been detected in over 127 countries. A recent study indicated that the Omicron’s progenitor might experience continuous evolution in mice and rapidly accumulate mutations to spill back to humans^4,5^.

As the spike glycoprotein of SARS-CoV-2 mediates the virus entry into host cells, it is the main target of NAbs and the hotspot for designing therapeutic agents and vaccines^6^. Omicron carries the most significant number of mutations among all SARS-CoV-2 variants to date, with 37 mutations within its spike protein, 15 of which are positioned at the RBD and cover almost all the critical mutation sites carried by previous VOCs, such as K417N, S477N, E484K (E484A in Omicron), and N501Y. These four substitutions have been reported to relate to immune escape and receptor binding^7–14^. The biological functions of emerging mutations of the Omicron spike need to be understood as a matter of urgency.

Recent data suggest that the Omicron spike or RBD engages human ACE2 (hACE2) with a similar or slightly increased affinity compared to the prototype^15–17^. Furthermore, Omicron significantly escapes from the immune protection elicited by natural COVID-19 infection and vaccination^18–21^. Most NAbs, including those for emergency use authorization (LY-CoV016, LY-CoV555, REGN10933, REGN10987, AZD1061, AZD8895, and BRII-196), show significant reductions or complete loss of neutralization potency except for VIR-7831 and DXP604^19,21,22^. VIR-7831 (sotrovimab) is derived from S309, identified from the memory B cells of individuals infected with SARS-CoV and can potently neutralize both SARS-CoV-2 and SARS-CoV^6^. According to the latest grouping scheme that categorizes RBD-targeting antibodies into seven major communities (RBD-1 to RBD-7), S309 belongs to the RBD-5 community targeting the outer face of RBD^23^. Except for those targeting the outer face of RBD (RBD-4 and −5 communities), antibodies recognizing the inner face of RBD (RBD-6 and −7 communities) are often cross-reactive and less vulnerable to immune escape by targeting more conserved epitopes. S304, one of the RBD-7 antibodies, has been reported to target a cryptic epitope buried in the spike’s closed state and cross-reacts with SARS-CoV-2 and SARS-CoV^24^. Its neutralizing capacity against the Omicron variant remains to be explored.

Investigating the characteristics of the Omicron spike and the impact of its mutations on binding ACE2 and NAbs is critical for understanding its high transmission and immune evasion. Although several groups have reported the structures of apo spike trimer of Omicron and its complexes with the ACE2 receptor or antibodies^15,17,25–27^, there are some controversies regarding spike conformational dynamics, and many questions remain to be answered. Here, we determined the structures of the apo, hACE2-bound or S309-bound spike trimer of Omicron, the ternary Omicron RBD-hACE2-S304 complex, and the hACE2-bound Omicron spike with three reverse mutations (L371S, P373S, and F375S) by cryo-EM. These results expand our understanding of the properties of the Omicron spike, shedding light on the potential molecular basis for the high transmission and immune evasion of Omicron.

## Results

### Omicron spike preferentially adopts the one-RBD-up conformation

**Fig. 1:**
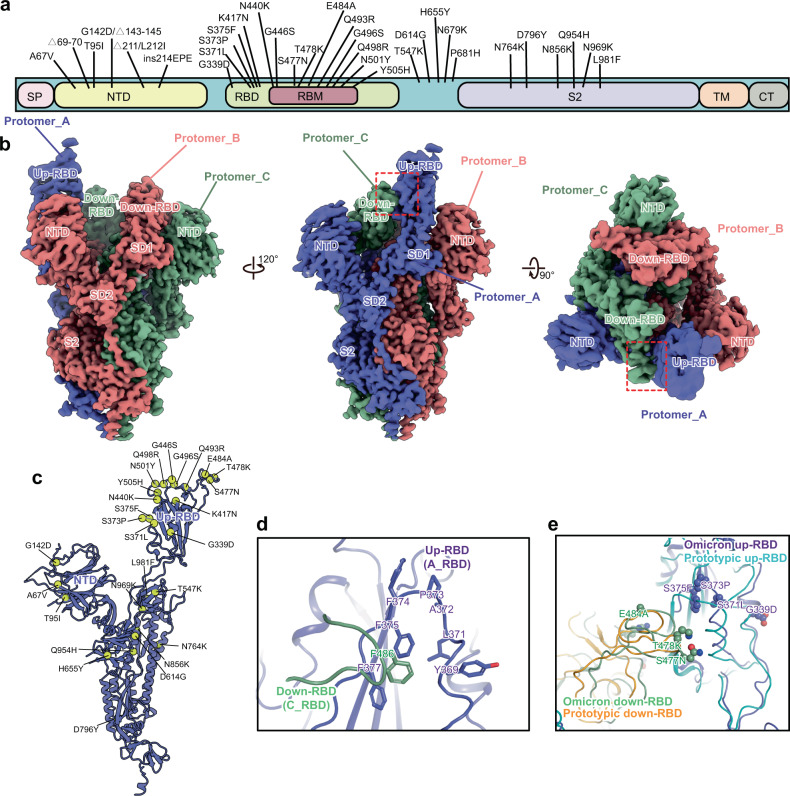
Cryo-EM structure of the Omicron spike ectodomain trimer. **a** A schematic diagram showing the amino acid mutations of the spike protein. **b** Cryo-EM map of the Omicron spike protein. Three protomers are colored in medium slate blue, light coral and dark sea green, respectively. The contact interface between down-and up-RBDs is boxed by the red dash line **c** Atomic structure of the Protomer_A of the Omicron spike with mutations shown as yellow spheres. **d** Contact interface of the Omicron up-RBD (Protomer_A, A_RBD) and neighboring down-RBD (Protomer_C, C_RBD). **e** Conformational comparison of the interface between A_RBD and C_RBD of the prototype (PDB: 6ZGG)^28^ and Omicron. Mutations of Omicron are shown as spheres.

The Omicron spike has 37 mutation sites, 11 located on the N-terminal domain (NTD), 15 on the RBD, and the remaining 11 near the furin cleavage site and on the S2 subunit (Fig. 1a). To explore the impact of emerging mutations on the structure and function of the spike protein, we determined the atomic structure of the apo Omicron spike at 3.03 Å resolution using single-particle cryo-EM (Fig. 1b, Supplementary Fig. 1 and Supplementary Table 1). The apo spike preferentially adopts the one-RBD-up open conformation (Supplementary Fig. 1). Its overall organization is similar to the prototype and other VOCs (Fig. 1b and Supplementary Fig. 2). Most mutations of the RBD and S2 are located near the interface between protomers (Fig. 1c). The subunit with the up-RBD was designated Protomer_A, and the other two subunits with the down-RBD were defined as Protomer_B and Protomer_C in a counterclockwise direction (Fig. 1b). A contact interface between the up-RBD (Protomer_A, A_RBD) and down-RBD (Protomer_C, C_RBD) was observed (Fig. 1d and Supplementary Fig. 3a–c). The interface is maintained by a hydrophobic microenvironment, where residue F486 of the C_RBD is closely surrounded by hydrophobic residues of the A_RBD and forms extensive hydrophobic contacts (Fig. 1d), consistent with previous observations^15,27^. Residue F486 of the C_RBD stacks with F375 of the A_RBD, bridging the up-RBD with the adjacent down-RBD. Superimposition of the Omicron spike with that of the prototype (PDB: 6ZGG)^28^ reveals that the interface between down- and up-RBDs of the Omicron spike adopts apparently different local conformations stretching to closer proximity compared to the prototype (Fig. 1e). These adaptations may enhance the stability of interchain interactions within the spike trimer and may further influence the conformational dynamics of RBDs.

### Omicron spike maintains the one-RBD-up conformation after ACE2 binding

**Fig. 2:**
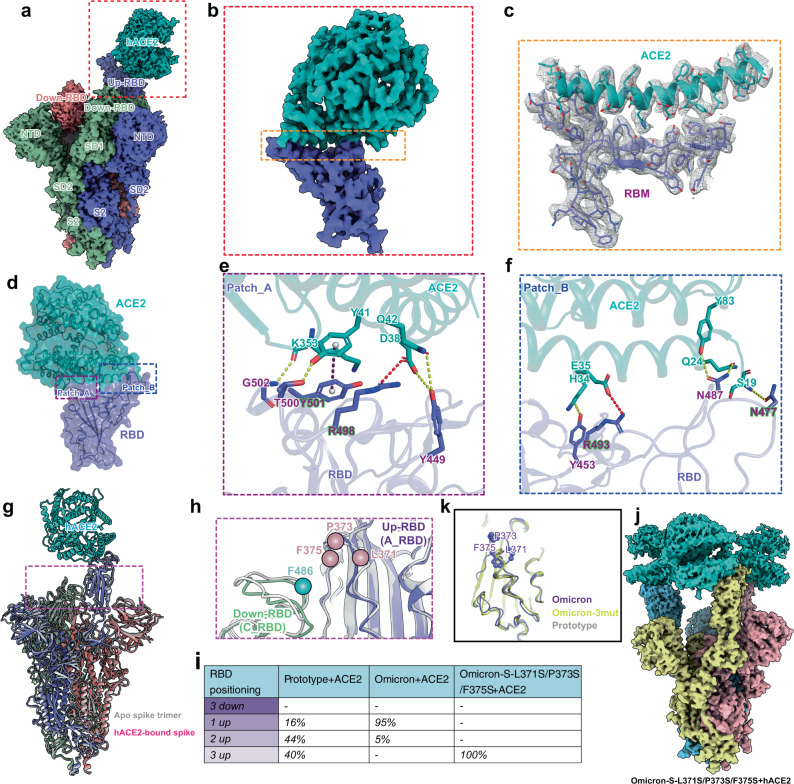
The architecture of the ACE2-bound spike trimer of Omicron. **a** Cryo-EM map of the one-RBD-up conformation of the Omicron spike protein in complex with hACE2 at 2.40 Å resolution. The ACE2 receptor is colored in cyan, and three protomers of the spike are colored corresponding to Fig. 1b. The inset box corresponds to the RBD-hACE2 region used for local refinement. **b** Density map of the Omicron RBD-hACE2 region at 3.24 Å resolution after local refinement for the boxed region in **a**. **c** Density map of the Omicron RBD-hACE2 interface with the fitted atomic model. Residues are shown as sticks, and density is represented in mesh. **d**–**f** Detailed interactions between RBD and hACE2. Yellow, red, and purple dashed lines represent hydrogen bonds, salt bridges and π-π interactions, respectively. **g** Superimposition of the apo and ACE2-bound spike trimers of Omicron that are reconstructed from the density maps at 3.03 Å and 2.85 Å resolution, respectively. **h** Conformational comparison of the interface between A_RBD and C_RBD in the apo and ACE2-bound spike trimers of Omicron. The apo spike trimer is colored in gray, and the C_RBD and A_RBD of the ACE2-bound spike trimer are colored in dark sea green and medium slate blue, respectively. The locations of residues F486 of the C_RBD, L371, P373 and F375 of the A_RBD are labeled with balls. **i** The relative population of RBD states observed in cryo-EM structures of ACE2-bound spikes for the prototype^30^, Omicron and Omicron-S-L371S/P373S/F375S mutant. **j** Cryo-EM map of the three-RBD-up conformation of the Omicron-L371S/P373S/F375S mutant in complex with hACE2 at 3.20 Å resolution. The ACE2 receptor is colored in cyan, and three protomers of the spike trimer are colored in khaki, light pink, and sky blue, respectively. **k** Alignment of the RBDs from the Omicron, Omicron-3mut (Omicron-S-L371S/P373S/F375S mutant), and the prototype (PDB: 6LZG)^41^. The RBDs of Omicron, Omicron-3mut, and the prototype are colored in purple, yellow and gray, respectively.

Previous data revealed that the spike undergoes conformational alteration by the rigid-body rotation of the RBD with a smaller conformational change in other domains of S1 components upon ACE2 binding^29,30^. The apo spike trimer of the prototype preferentially adopts two conformations: all-down and one-RBD-up conformations, with near equivalent proportions (Supplementary Fig. 2)^31^. The apo spike of the Beta and Delta variants also preferentially adopts all-down or one-RBD-up conformation with a higher ratio of the one-RBD-up conformation than the all-down conformation (Supplementary Fig. 2)^32,33^. Two-RBD-up conformation was observed in the apo spike of the D614G strain and Alpha VOC (Supplementary Fig. 2)^32,34^. For the Gamma VOC, the one-RBD-up conformation of its spike was observed (Supplementary Fig. 2)^33^. When bound to ACE2, the prototypic spike preferentially adopts two- and three-RBD-up conformations (Supplementary Fig. 4)^30^. Although the previous four VOCs (Alpha, Beta, Gamma, and Delta) harbor different mutations in their spikes, all of these spikes undergo significant conformational alteration after ACE2 binding, similar to the prototype (Supplementary Figs. 2, 4)^32,33,35^. To explore the impact of ACE2 binding on the Omicron spike, we mixed soluble Omicron spike trimer (Supplementary Fig. 5a) with monomeric hACE2 at a 1:4 molar ratio and incubated it overnight at 4 °C. Excessive hACE2 was removed by consecutive diluting with 20 mM Tris (pH 8.0) and 150 mM NaCl and concentrating using a centrifugal concentrator. Single-particle cryo-EM data were then collected, and the atomic structure of the hACE2-bound Omicron spike was determined. The Omicron spike in complex with hACE2 preferentially adopts the one-RBD-up open state with a prevalence of 95% compared to the two-RBD-up conformation at a 5% ratio (Fig. 2a and Supplementary Fig. 6), which is significantly different from the prototype and other VOCs (Supplementary Fig. 4).

We then performed local refinement focusing on the RBD-hACE2 binding region and obtained a density map at 3.24 Å resolution (Fig. 2b, Supplementary Fig. 6, and Supplementary Table 1), which clearly defined the amino acid side chains at the RBD-hACE2 interface (Fig. 2c). Structural analysis revealed that substitutions of S477N, Q493R, and Q498R form hydrogen bonds or salt bridges with S19, E35 and D38 on hACE2, respectively (Fig. 2d–f). The N501Y mutation establishes a π–π interaction with Y41 of ACE2 (Fig. 2e), similar to that observed in the Alpha and Beta variants^14,36–39^. The K417N substitution abolishes the salt bridge to D30, consistent with previous studies^14,36,40^.

To visualize the interface between the down- and up-RBDs of the hACE2-bound spike, we performed the RBD-focused three-dimensional (3D) classification and obtained a density map at 2.85 Å resolution (Supplementary Fig. 6). Superimposition of one-RBD-up conformation of the spike-hACE2 complex and apo spike of Omicron revealed that the one-RBD-up state has no apparent conformational changes after ACE2 binding (Fig. 2g). The interface of the down- and up-RBDs is near the ACE2-binding region and well maintained after ACE2 binding (Fig. 2h and Supplementary Fig. 3d–f). To test whether the three substitutions of Omicron (S371L, S373P, and S375F) are critical for its stable one-RBD-up conformation, we generated Omicron-S-L371S/P373S/F375S mutant protein with the three residues (L371, P373, and F375) replaced by those (S371, S373, and S375) in the prototypic spike. The purified mutant protein was incubated with hACE2 in the same way to determine the cryo-EM structure. Strikingly, the structure revealed that the mutant spike predominantly exists in the three-RBD-up conformation after ACE2 binding (Fig. 2i, j), indicating that these three reverse mutations prime to transform into open conformation when bound to the ACE2 receptor. Local refinement for the RBD-hACE2 region of the complex was performed, and a clear density map was obtained (Supplementary Fig. 7). Structural alignment showed that residues 363-380 of the mutant protein have an obvious conformational change compared to that of the Omicron RBD and adopts the same conformation as the prototype (PDB: 6LZG)^41^ (Fig. 2k), indicating that the conformational change in the region (residues 363-380) observed in Omicron is attributable to these three substitutions (S371L, S373P, and S375F).

### Structure of the S309-bound Omicron spike trimer

**Fig. 3:**
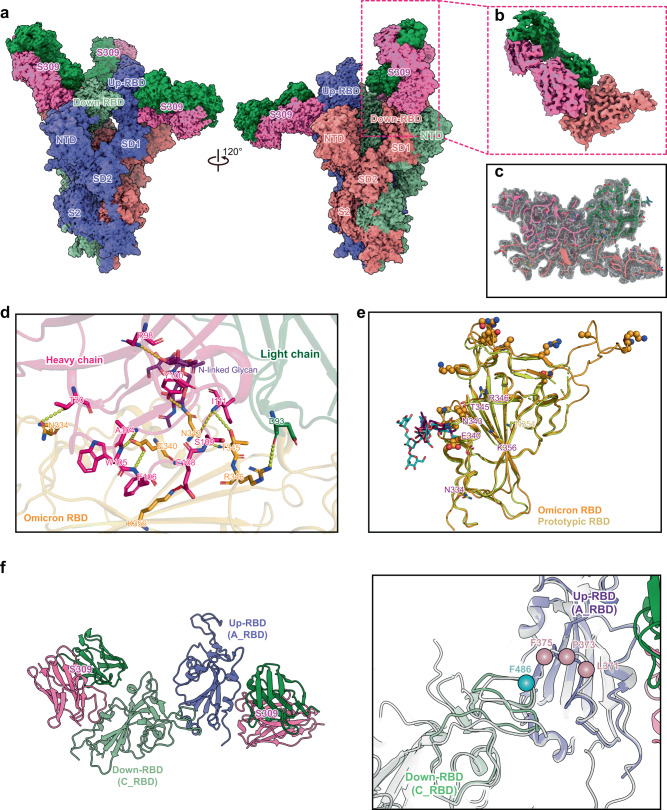
Cryo-EM structure of the Omicron spike-S309 complex. **a** Cryo-EM map of the one-RBD-up conformation of the Omicron spike protein in complex with three S309 Fabs at 2.50 Å resolution. Three protomers of the spike are colored corresponding to Fig. 1b. The heavy and light chains of the S309 Fab are colored in hot pink and forest green. The inset box corresponds to the RBD-S309 region used for local refinement. **b** Cryo-EM map of the RBD-S309 region at 2.80 Å resolution after local refinement for the boxed region in **a**. **c** Density map of the Omicron RBD-S309 interface with the fitted atomic model. Residues are shown as sticks, and density is represented in mesh. **d** Detailed interactions between Omicron RBD (orange) and S309 Fab (hot pink and forest green). The N-linked glycan of N343 in the RBD is colored in purple. Yellow and red dashed lines represent hydrogen bonds and salt bridges, respectively. **e** Comparison of key residues of the epitope targeted by S309 in the prototype (yellow) (PDB: 6WPT)^6^ and Omicron (orange). Key residues shared by the prototype and Omicron are labeled in purple, otherwise are labeled with their respective colors. Mutations of the Omicron RBD are shown as spheres. N-linked glycan of the residue N343 in the prototype or Omicron is colored in cyan or purple. **f** The structure of the S309-RBD-RBD-S309 region at 2.66 Å resolution after local refinement (left panel). The colors shown correspond to **a**. The interfaces between up-RBD (A_RBD) and down-RBD (C_RBD) in the apo and S309-bound Omicron spikes were compared (right panel). The locations of residues F486 of the C_RBD, L371, P373, and F375 of the A_RBD are labeled with balls.

To shed light on the molecular basis for S309 retaining its neutralizing activity against Omicron, we expressed the S309 antibody^6^ and prepared the spike-S309 complex as used in the spike-hACE2 complex. The S309-bound Omicron spike trimer was then determined by single-particle cryo-EM (Supplementary Fig. 8). The one-RBD-up conformation of the spike-S309 complex accounts for the most significant proportion at over 82% (Fig. 3a and Supplementary Fig. 9). Focused refinement for the RBD-S309 region was further performed (Fig. 3b and Supplementary Fig. 8). The density map at 2.80 Å resolution allows us to visualize the interface of RBD and S309 Fab (Fig. 3c, Supplementary Fig. 8, and Supplementary Table 1). S309 targets a proteoglycan epitope on N343 of RBD and its complementary determining regions loops form extensive hydrogen bonds with residue N334, E340, N343, T345, R346, and K356 of the Omicron RBD (Fig. 3d), similar to that in the prototype (PDB: 6WPT)^6^. These key residues targeted by S309 are not significantly affected by the emerging mutations of the Omicron spike (Fig. 3e). In addition, the epitope is positioned in the outer face of the RBD and is accessible in both up and down configurations of the RBD (Fig. 3a). These two features make S309 retain binding and neutralizing capacities for the Omicron variant. The S309-RBD-RBD-S309 region was also performed local refinement, and a density map at 2.66 Å resolution was obtained (Supplementary Fig. 8 and Supplementary Table 1). The structure showed that the interface between the down- and up-RBDs is maintained after S309 binding (Fig. 3f and Supplementary Fig. 3g–i).

### Molecular mechanism of the reduced neutralizing capacity of the S304 antibody against Omicron

**Fig. 4:**
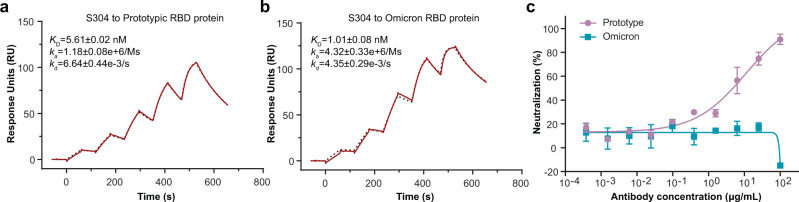
Binding affinity and neutralizing capacity of S304 for the Omicron variant. **a**, **b** The S304 antibody was captured on a Protein A chip and then tested for binding with gradient concentrations of the prototypic RBD (3.125 nM, 6.25 nM, 12.5 nM, 25 nM and 50 nM) (**a**) and Omicron RBD (1.5625 nM, 3.125 nM, 6.25 nM, 12.5 nM, and 25 nM) (**b**) in a single-cycle mode. The binding profiles are shown with time (s) on the *x*-axis and response units (RU) on the *y*-axis. The black dotted curves were obtained by fitting data to the 1:1 binding model (Biacore Insight Evaluation software v.3.0). *K*_D_, *k*_a_, and *k*_d_ values shown are the mean ± standard deviation (SD) of three independent experiments. **c** VSV-based pseudotyped virus neutralization assay. The prototype and Omicron pseudoviruses were incubated with fourfold serial dilutions of S304 NAb, respectively. The mixture was then added to Vero cells. After 15 h, the infected cells were counted using a CQ1 confocal image cytometer (Yokogawa). Three independent experiments were performed with two replicates. The curves and IC_50_ values are one representative data, in which the error bar for each concentration is presented as mean ± SD. Source data are provided as a Source Data file.

S304 is a weak NAb isolated from a patient with convalescent SARS, which cross-reacts with SARS-CoV-2 and SARS-CoV^6^. S304 recognizes the inner face of the RBD buried in the down RBD configuration^23^; therefore, its binding to the spike is conformation-dependent. Surface plasmon resonance (SPR) analysis suggested that the binding affinity of S304 for the Omicron RBD (*K*_D_ = 1.01 nM) is slightly higher than that of the prototypic RBD (*K*_D_ = 5.61 nM) (Fig. 4a, b). However, its neutralizing activity against Omicron (IC_50_ > 100 μg/mL) is dramatically impaired relative to that of the prototype (IC_50_ = 10.94 μg/mL) (Fig. 4c).

**Fig. 5:**
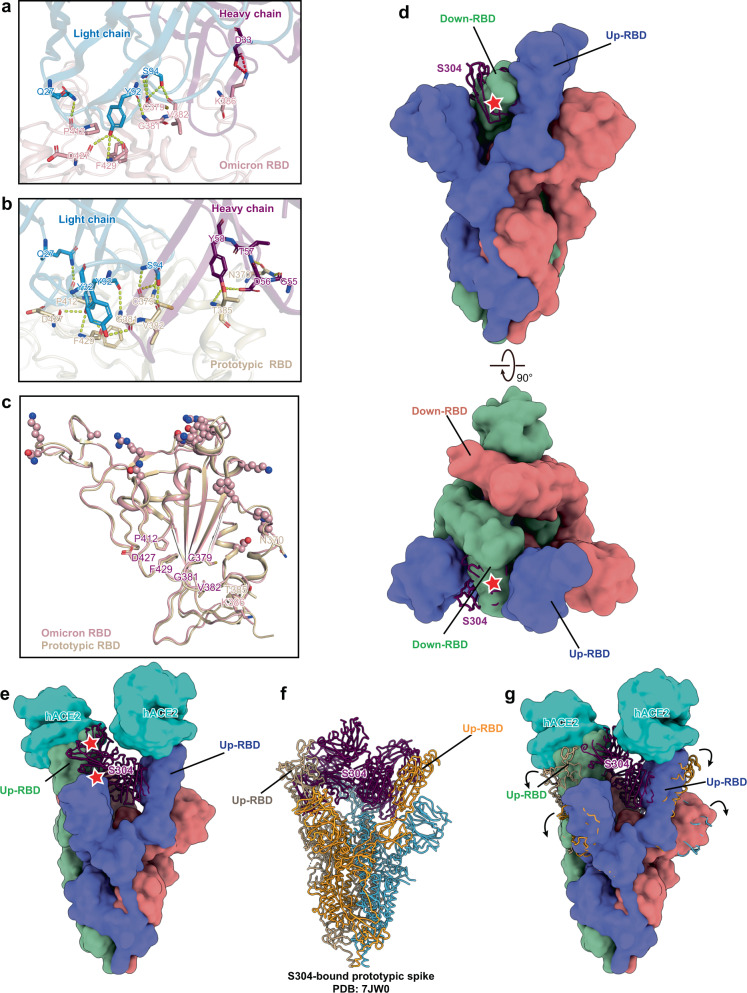
One-RBD-up conformation of Omicron spike impairs the binding and neutralizing potencies of S304 antibody. **a** Detailed interactions between Omicron RBD (pink) and S304 Fab (blue and purple). Yellow and red dashed lines represent hydrogen bonds and salt bridges, respectively. **b** Interaction analysis between prototypic RBD (tan) and S304 Fab (blue and purple) in the RBD-S304-S309-S2H14 complex (PDB: 7JX3)^24^. Yellow and red dashed lines represent hydrogen bonds and salt bridges, respectively. **c** Comparison of key residues of the epitope targeted by S304 in the prototype (tan) (PDB: 7JX3)^24^ and Omicron (pink). Key residues shared by the prototype and Omicron are labeled in purple, otherwise are labeled with their respective colors. Mutations of the Omicron RBD are shown as spheres. **d** Superimposition of the S304-bound Omicron RBD (purple) on the apo spike trimer of Omicron. Three protomers of which are colored corresponding to Fig. 1b. The star shape represents the clash site. **e** Superimposition of the S304-bound Omicron RBD on the ACE2-bound spike trimer of Omicron with two-RBD-up conformation revealed that the steric clash between S304 and the neighboring RBD and NTD remained. **f** The atomic structure of three S304-bound spike trimer of the SARS-CoV-2 prototype (PDB: 7JW0)^24^. **g** Superimposition of the model in **f** on the two ACE2-bound Omicron spike.

To illustrate the mechanism of its reduced neutralizing activity, we purified the Omicron RBD-hACE2-S304 complex (Supplementary Fig. 5b) and determined its atomic structure (Supplementary Fig. 10 and Supplementary Table 1). We did not build the model for the constant domain of S304 Fab because its density is less visible in our structure (Supplementary Fig. 10). Structural analysis indicated that the epitope targeted by S304 is conserved. The contact network contributed by RBD’s residues C379, G381, V382, P412, D427, and F429 is maintained well (Fig. 5a, b). Residue N370 and T385 formed hydrogen bonds with G55, D56, T57, and Y58 of the S304 heavy chain in the prototype (PDB:7JX3)^24^ (Fig. 5b). In Omicron, the hydrogen bond network formed by N370 and T385 of the RBD is replaced by two salt bridges between residue K386 of RBD and D33 of the S304 heavy chain (Fig. 5a). Except for N370, other key residues targeted by S304 are far from the mutation sites, and their conformations are not significantly affected by these substitutions (Fig. 5c). Combined with the SPR results (Fig. 4a, b), these changes do not reduce the binding affinity of S304 for Omicron RBD.

The apo spike trimer of the prototype shows all-down and one-RBD-up conformations, with no two- or three-RBD-up conformations (Supplementary Fig. 2). More conformations were observed in the mixture of the prototypic spike and ACE2, including the two-RBD-up conformation of the apo spike trimer, with a prevalence of 4%^29^. The three-RBD-up conformation of the apo prototypic spike trimer has not yet been observed. Because the epitope of S304 is located at the inner face of the RBD, S304 could not directly bind to the one-RBD-up conformation of the prototypic spike. However, the S304 antibody could neutralize the prototype (Fig. 4c). In addition, the structure of the S304-bound prototypic spike showed that the spike binds three S304 Fabs in a three-RBD-up conformation^24^. Therefore, the S304 antibody may bind to the spike in a “special” manner. The intermediate conformation with disordered RBD densities and the motion of RBD^28,36^ suggest a dynamic conformational alteration of RBD^42,43^. This motion and dynamics of the virus envelope proteins have been reported in many enveloped viruses, such as influenza^44,45^_,_ dengue^46^, human immunodeficiency^47^, and respiratory syncytial^48^ viruses. S304 may bind to the spike trimer during the motion of RBDs. However, the Omicron spike packs more tightly than in the prototype^15^, and the contact interface between down- and up-RBDs helps stabilize the one-RBD-up conformation (Fig. 1d). The fact that both apo and ACE2-bound Omicron spikes preferentially adopt the one-RBD-up conformation suggests that the conformational change from down- to up-RBDs of Omicron may require more energy than the prototype, whose spike preferentially adopts the two- and three-RBD-up conformations after binding the ACE2 receptor (Supplementary Fig. 4). These results support the notion that Omicron RBD’s motion is weaker than that of the prototype.

The Superimposition of S304-bound Omicron RBD (Supplementary Fig. 10) into the apo spike with the one-RBD-up conformation (Fig. 1b) showed severe steric clashes between S304 and the down-RBD (Fig. 5d). The S304 Fab from the S304-bound prototypic spike (PDB: 7JW0)^24^ was aligned with our Omicron RBD-S304 structure and these two structures were further superimposed into the Omicron spike with the two-RBD-up conformation from the Omicron spike-hACE2 complex, which revealed that the steric clash between the S304 and the up-RBD and NTD still exists (Fig. 5e). The structure of the prototypic spike bound to S304 (PDB: 7JW0)^24^ and alignment with the Omicron spike with two-RBD-up conformation showed that elimination of the steric hindrance requires RBDs and NTDs of the Omicron spike to shift outward (Fig. 5f, g). Due to the reduced motion of the Omicron RBDs, this outward shift could be suppressed. Taken together, the more stable one-RBD-up state of the Omicron spike reduces the chance of exposing the epitope of S304 NAb, thus resulting in compromised neutralizing efficiency.

## Discussion

The rapid spread of Omicron has caused widespread concern. The mechanisms resulting in the high transmission and severe immune evasion of the Omicron variant are not well understood. During our manuscript preparation, Mannar et al. determined the structure of the apo spike trimer of Omicron, in which only one RBD in a down configuration is well-resolved, while the other two RBDs are less visible because of their flexibility^17^. After binding ACE2, only two-RBD-up conformation was observed^17^. Another group found that the apo spike trimer of Omicron only adopts one-RBD-up conformation in the neutral and endosome pH, and a unique interface between up- and down-RBDs underpins the up configuration^15^. After binding ACE2, two-RBD-up conformation was found^15^, in agreement with Mannar et al.^17^. However, Yin et al. isolated the all-down conformation of the apo spike trimer at a high percentage (70%) from all picked particles of the Omicron spike-hACE2 complex^27^. The rest (30%) adopted a one-RBD-up conformation binding to hACE2, with no two-RBD-up conformation found^27^. To visualize the interface between the down- and up-RBDs, they performed local refinement for the RBD-RBD-hACE2 region (Supplementary Fig. 3j–l)^27^. Three RBDs in the all-down conformation are less visible in contrast to the clear visibility of three RBDs in the spike-ACE2 complex, suggesting that the RBDs in the apo form are dynamic, and ACE2 binding might stabilize the conformations of three RBDs^27^. In our study, the one-RBD-up conformation predominates in the apo, ACE2-, or S309-bound spike trimers. Furthermore, our group resolved the cryo-EM structure of the mouse ACE2-bound Omicron spike, which also adopts the one-RBD-up conformation^49^. These results indicate that the conformational changes from the down-RBD to the up-RBD of Omicron may be reduced, even adding the ACE2 receptor. The stable one-RBD-up conformation impairs the neutralization potency of S304. Therefore, enhanced stability of one-RBD-up conformation may be another mechanism for SARS-CoV-2 to escape from the conformationally selected antibodies. Due to the striking immune escape demonstrated by Omicron, finding pan-sarbecovirus NAbs targeting conserved and conformation-independent epitopes may be a future direction for therapeutic NAbs. In addition, the three-RBD-up conformation of hACE2-bound mutant Omicron spike with reverse mutations (L371S, P373S and F375S) reveals the three emerging substitutions of Omicron (S371L, S373P, and S375F) play a crucial role in stabling the one-RBD-up conformation of the spike to fight against the conformational alteration triggered by ACE2 binding. The predominantly one-RBD-up conformation of the Omicron spike is consistent with recent reports during our manuscript revision^15,50,51^ Given a large number of emerging mutations on the Omicron spike, the impact of other mutations on the spike’s conformation deserved further study.

Worker ants and worker bees have an amazing ability to communicate with each other and work together to accomplish complex tasks. In the Omicron SARS-CoV-2, one-RBD-up conformation is preferred due to key amino acid mutations for the interactions of the other RBDs in one trimeric molecule. Like in the bee or ant society, collaborative teamwork is the key to accomplishing complex tasks with, sometimes, the sacrifice of a small group’s life. In this case, the other two RBDs keep their down conformation to help the up one easily approach the ACE2 receptor. So here, we propose a “teamwork” effect model for the Omicron variant to balance the receptor binding and immune escape.

Omicron has recently been subdivided into three lineages (BA.1, BA.2, and BA.3). BA.2 has rapidly replaced BA.1 as the dominant sub-variant. BA.2 has nearly completely distinct substitutions in the NTD region compared to BA.1, implying the reconstruction of the NTD region. Some NTD-targeting protective antibodies for BA.1 may not protect from BA.2. However, the RBD of BA.2 has many common mutations with BA.1 accompanied by an increase in three new substitutions (T376A, D405N, and R408S) and the loss of two mutation sites (G446S and G496S). D405 and R408 are key residues of the broadly NAbs H014 and S2A4. Substitutions of D405N and R408S may influence the binding and neutralization potencies of these NAbs. G446S was thought to be an important immune escape site for REGN-10987^15^. The loss of the G446S substitution in BA.2 implies that REGN-10987 maintains its binding affinity and neutralizing potency for BA.2. The functional characteristics of the BA.2 spike deserve further assessment in future studies.

## Methods

### Gene construction

The Omicron spike ectodomain protein (Novoprotein, Cat: DRA193) with furin cleavage site mutants (R682G, R683S, and R685S), 2P mutants (K986P and V987P), a C-terminal T4 fibritin trimerization domain and a 6× His tag, referred to as S-2P-GSAS, was purchased. Gene encoding the ectodomain of the Omicron spike protein (residues 1-1208, GISAID: EPI_ISL_6590782.2) with 6P mutants (F817P, A892P, A899P, A942P, K986P, and V987P) and the reserved furin cleavage site (_682_RRAR_685_), referred to as S-6P-RRAR, was also synthesized by GENEWIZ. It was fused with a C-terminal T4 fibritin trimerization domain, a Strep-tag II, and an 8× His tag and cloned into a mammalian cell expression vector pCAGGS. Reverse mutations (L371S, P373S, and F375S) were introduced into the S-6P-RRAR to obtain the S-L371S/P373S/F375S construction. The variable region of NAbs, S309, and S304, fused with the constant region of immunoglobulin G1, was synthesized and cloned into pCAGGS vectors^6^. The coding sequence of hACE2 (residues 19-615, GenBank: NP_001358344) tagged with a C-terminal 8× His tag was cloned into the pCAGGS expression vector containing an N-terminal IL-10 signal peptide. The coding sequence of the SARS-CoV-2 RBD (residues 319-541, GISAID: EPI_ISL_402119), tagged with a C-terminal 6× His tag, was cloned into the pCAGGS expression vector.

### Protein expression and purification

The spike protein was expressed in HEK293F cells grown in suspension in SMM 293-TII medium (Sino Biological, Cat# M293TII) at 37 °C in a humidified 5% CO2 incubator rotating at 130 rpm. The cells were transfected with a mixture of 1 g Omicron spike expression plasmid and 3 mg polyethyleneimine at a density of 2 × 10^6^ cells/mL. A supplement (Sino Biological, Cat# M293-SUPI) was added to the culture system 24 h after transfection. Approximately 60 h after transfection, the cells were removed via centrifugation for 2 h at 6500 *g*. The supernatant was purified using a HisTrap HP 5 mL column (GE Healthcare) using metal affinity chromatography. Proteins were eluted using buffer containing 20 mM Tris (pH 8.0), 150 mM NaCl, and 300 mM imidazole. The eluted product was purified via affinity chromatography using a StrepTrap HP 5 mL column (GE Healthcare). Proteins were eluted using a buffer containing 20 mM Tris (pH 8.0) and 150 mM NaCl supplemented with 5 mM d-desthiobiotin. The proteins were further purified by gel filtration chromatography using a HiLoad Superose® 6 increase 10/300 GL (GE Healthcare) with a running buffer of 20 mM Tris (pH 8.0) and 150 mM NaCl.

S309, S304, SARS-CoV-2 RBD, and hACE2 were expressed in HEK293F cells. The culture supernatants of HEK293F cells were collected 5 days post-transfection and purified using a Protein A 5 mL affinity column (GE Healthcare) for the S309 and S304 antibodies or by a HisTrap HP 5 mL affinity column (GE Healthcare) for SARS-CoV-2 RBD and hACE2. These soluble proteins were further purified using gel filtration with a Superdex™ 200 10/300 GL column (GE Healthcare). S309 and S304 Fabs were generated via papain digestion and further purified using a Protein A column (GE Healthcare) and gel filtration with a Superdex^TM^ 200 10/300 GL column (GE Healthcare).

### Protein complex formation

For the ACE2-bound or S309-bound Omicron spike and ACE2-bound Omicron spike with L371S, P373S, and F375S mutations, the spike protein was incubated with the purified hACE2 at a 1:4 molar ratio (spike trimer to hACE2) overnight at 4 °C before purification by concentration and dialysis using a 100-kDa cut-off Ultracon concentrator (Millipore) with 20 mM Tris (pH 8.0) and 150 mM NaCl. For the Omicron RBD-hACE2-S304 complex, RBD and hACE2 were co-incubated for 2 h at 4 °C at a 1:1.2 molar ratio. The mixture was then purified via gel filtration using a Superdex^TM^ 200 10/300 GL (GE Healthcare) to obtain the Omicron RRD-hACE2 complex. The Omicron RBD-hACE2 and S304 Fab were then mixed at a 1:1.5 molar ratio and incubated overnight at 4 °C, followed by gel filtration using a Superdex^TM^ 200 10/300 GL (GE Healthcare) to obtain Omicron RBD-hACE2-S304 complex. The protein complex was stored in a buffer containing 20 mM Tris-HCl (pH 8.0) and 150 mM NaCl for cryo-EM sample preparation.

### Cyro-EM sample preparation and data acquisition

For the apo Omicron spike trimer, C-flat R1.2/1.3 (300 mesh) holey carbon grids were first glow discharged for 12 s using a Pelco easiGlow glow discharge unit, and 3 µl protein at a concentration of 1.0 mg/mL was applied to the surface of the grid at a temperature of 10 °C and a humidity level of 100%. The grids were then blotted for 2 s before being plunge-frozen in liquid ethane using Vitrobot Mark IV (Thermo Fisher Scientific). Grids were imaged using a 300 kV Titan Krios electron microscope (Thermo Fisher Scientific) equipped with a K3 direct electron detector in the super-resolution counting mode. Movies were collected at 1.11 Å per physical pixel over a defocus range of −1.0 to −2.0 μm, with a total dose of 60 e^–^/Å^2^ using EPU automated acquisition software.

For the Omicron spike-hACE2 or S309 Fab complex, a droplet (3.0 μL) of the complex at a concentration of 3.0 mg/mL was applied to glow-discharged C-flat R1.2/1.3 (300 mesh) holey carbon grids and subsequently vitrified using Vitrobot Mark IV (Thermo Fisher Scientific). Cryo-EM movie stacks were collected using Titan Krios microscopes operated at 300 kV in energy-filtered transmission electron microscopy mode. A nanoprobe with a 0.85-μm illumination area was used. Data were recorded by a K3 direct summit camera at a nominal magnification of 130,000 using a super-resolution counting model at a physical pixel size of 0.66 Å (Omicron spike-hACE2 complex) or 0.67 Å (Omicron spike-S309 Fab complex). A BioQuantum energy filter was operated in zero-energy-loss mode with an energy slit width of 20 eV. The total accumulative electron dose was 50 e^–^/Å^2^ fractioned over 32 subframes, with a total exposure time of 1.2 s. The target defocus range was set to −1.0 to −2.5 μm.

For the hACE2-bound Omicron spike trimers with reverse mutations (L371S, P373S, and F375S), C-flat R1.2/1.3 (300 mesh) holey carbon grids were first glow discharged for 12 s using a Pelco easiGlow glow discharge unit, and 3 µl protein at a concentration of 3.0 mg/mL was applied to the surface of the grid at a temperature of 10 °C and a humidity level of 100%. The grids were then blotted for 2 s before being plunge-frozen in liquid ethane using Vitrobot Mark IV (Thermo Fisher Scientific). Grids were imaged using a 300 kV Titan Krios electron microscope (Thermo Fisher Scientific) equipped with a K3 direct electron detector in the super-resolution counting mode. Movies were collected at 0.88 Å per physical pixel over a defocus range of –1.0 to –2.0 μm, with a total dose of 50 e^–^/Å^2^ using EPU automated acquisition software.

For the Omicron RBD-hACE2-S304 complex, Quantifoil R1.2/1.3 (Au 300 mesh) holey carbon grids supported with a thin layer of graphene oxide were first glow discharged for 20 s using a HARRICK PLASMA PDC-32G-2 glow discharge unit, and 4 µl protein was applied to the surface of the grid at a temperature of 8 °C and a humidity level of 100%. The grids were then blotted for 1.5 s before being plunge-frozen in liquid ethane using Vitrobot Mark IV (Thermo Fisher Scientific). Grids were imaged using a 300 kV Titan Krios electron microscope (Thermo Fisher Scientific) equipped with a K3 direct electron detector in the super-resolution counting mode. Movies were collected at 0.669 Å per physical pixel over a defocus range of −1.0 to −2.0 μm, with a total dose of 50 e^–^/Å^2^ using EPU automated acquisition software.

### Image processing and 3D reconstruction

For the apo spike trimer, 7869 super-resolution movies were collected and corrected for drift using MotionCor2 v.1.4.2^52^. Subsequent image processing and reconstruction were performed using cryoSPARC v.3.3.1^53^. Contrast transfer function (CTF) parameters were determined using CTF estimation in the patch mode. Blob particle picking, particle extraction, and two-dimensional (2D) classification were performed on a subset of 200 micrographs to generate templates for auto-picking against the entire dataset. A total of 3,153,035 particles were selected from the 7869 micrographs. After extraction, these particles were used for 2D classification. A total of 1,023,001 particles were used for initial reconstruction and heterogeneous refinement. A total of 606,616 particles were used for the focused 3D classification based on the RBD mask. Finally, 98,093 particles were used for non-uniform refinement and iterative cycles of global and local CTF refinement, and a density map at 3.03 Å resolution was obtained. The map was sharpened by DeepEMhancer^54^.

For the Omicron spike-hACE2 complex, dose-fractionated image stacks were subjected to beam-induced motion correction using MotionCor2 v.1.4.2. The initial CTF values for each micrograph were calculated using CTFFIND-4.1^55^. Micrographs with an estimated resolution limit worse than 4.50 Å were discarded in the initial screening. A set of ∼100,000 particles was blob-picked and subjected to 2D classification to generate templates for auto-picking against the entire dataset. Subsequent image processing and reconstruction were performed using cryoSPARC v.3.3.1^53^ and RELION-3.1^56^. A total of 2,206,987 particles were selected from the 14,098 micrographs. The selected particles were then extracted and subjected to four rounds of reference-free 2D classification, yielding 1,298,852 projection particles. This subset was subjected to one round of heterogeneous refinement, and complex subsets were used to obtain a map with a resolution of 3.0 Å. This portion of the particle projections was imported to RELION-3.1 for further 3D classification. After 3D classification, two types of Omicron spike-hACE2 structures, one-RBD-up and two-RBD-up conformations, were identified. The particles with the one-RBD-up conformation were imported back to cryoSPARC v3.3.1, and the structure was obtained at 2.40 Å global resolution. Local refinement, focusing on the RBD-ACE2 with a mask, could reconstitute the RBD-ACE2 structure at 3.24 Å resolution. The particles with a two-RBD-up conformation were reconstructed, and a structure at 3.44 Å resolution was obtained. To improve the density map quality of the RBD-RBD region, we used particles obtained from the heterogeneous refinement to perform the RBD-focused 3D classification followed by homogeneous refinement that resulted to a density of Omicron spike bound to one hACE2 at 2.85 Å global resolution. The map was sharpened by DeepEMhancer^54^.

For the hACE2-bound spike trimer with reverse mutations (L371S, P373S, and F375S), 3395 super-resolution movies were collected and corrected for drift using MotionCor2 v1.4.2. Subsequent image processing and reconstruction were performed using cryoSPARC v3.3.1. Contrast transfer function (CTF) parameters were determined using CTF estimation in the patch mode. Blob particle picking, particle extraction, and 2D classification were performed on a subset of 1000 micrographs to generate templates for auto-picking against the entire dataset. A total of 7,582,944 particles were selected from the 3,395 micrographs. After extraction, these particles were used for 2D classification. A total of 170,172 particles were used for initial reconstruction and heterogeneous refinement. A total of 78,588 particles were used for the homogeneous refinement, and a structure of the three ACE2-bound spike at 3.20 Å global resolution was obtained. The RBD-hACE2 region was further performed local refinement and resulted in a density map at 3.11 Å resolution. Furthermore, particles from the heterogeneous refinement were performed 3D classification to identify different conformations.

The Omicron spike-S309 complex was processed similarly to the Omicron spike-ACE2 data. Briefly, 2,895,263 automatically picked particles were extracted from cryoSPARC v3.3.1 for the following 2D classification. Three rounds of reference-free 2D classification were performed. A dataset with 1,274,621 particles was selected and subjected to one round of heterogeneous refinement. The complex subsets were used to obtain a map at 2.9 Å resolution in cryoSPARC v3.3.1. This portion of particle projections was imported to RELION-3.1 for further 3D classification. After 3D classification, two types of Omicron spike-S309 fab structures were identified, including one-RBD-up and two-RBD-up conformations both of which bind three S309 Fabs. The particles with one-RBD-up conformation were imported back to cryoSPARC v3.3.1, and a structure was obtained at 2.50 Å global resolution. Local refinement, focused on the RBD-S309 region with a mask, could reconstitute the RBD-S309 structure at 2.80 Å resolution. To improve the density quality of the RBD-RBD region, we used particles from the heterogeneous refinement to perform the RBD-focused 3D classification followed by homogeneous refinement and local refinement that resulted to a density of S309-RBD-RBD-S309 at 2.66 Å resolution. The map was sharpened by DeepEMhancer^54^.

For the Omicron RBD-hACE2-S304 complex, 7899 super-resolution movies were collected and corrected for drift using MotionCor2 v1.4.2, and the CTF parameters were determined using CTFFIND-4.1. Subsequent image processing and reconstruction were performed using cryoSPARC v3.3.1. Blob particle picking, particle extraction, and 2D classification were performed on 7899 micrographs, resulting in 7,267,081 particles. After extraction and splitting, these particles were used in batch 2D classification. A total of 2,864,717 particles were used for initial reconstruction and heterogeneous refinement. A total of 1,635,544 particles were used for non-uniform refinement. The iterative cycles of the global CTF refinement and per-particle defocus refinement improved the map to 3.05 Å.

### Model building

The structures of the spike protein (PDB: 6VSB), spike-hACE2 complex (PDB: 7KNB), spike-S309 complex (PDB: 6WPS), and RBD-S304 region extracted from the RBD-S2E12-S309-S304 complex (PDB: 7R6X) were docked into the cryo-EM density maps of the apo spike, spike-hACE2, spike-S309 and Omicron RBD-hACE2-S304 complexes using Chimera v.1.14^57^, respectively. The models were manually corrected and refined iteratively using COOT v.0.9.8^58^ and PHENIX v1.19.2^59^. The stereochemical quality of each model was evaluated using MolProbity^60^. Structural figures were generated using PyMOL v.2.4^61^, Chimera v.1.14^57^, and ChimeraX v.1.3^62^.

### Measurement of S304 binding to Omicron and prototypic spike using SPR

The affinities and kinetics were analyzed using the BIAcore 8 K (GE Healthcare) performed at 25 °C in single-cycle mode. The proteins were transferred into phosphate-buffered saline/Tween 20 (PBST; 10 mM Na_2_HPO_4_, 2 mM KH_2_PO_4_, 137 mM NaCl, 2.7 mM KCl, pH 7.4, and 0.005% [v/v] Tween 20) buffer. The S304 NAb was captured on a protein A chip (GE Healthcare). Gradient concentrations of prototypic RBD from 50 nM to 3.125 nM and Omicron RBD from 25 nM to 1.5625 nM with twofold dilution flowed over the chip in PBST buffer. The Protein A chip (GE Healthcare) was regenerated using 10 mM Glycine-HCl (pH 1.5). The affinity values were calculated using a 1:1 (Langmuir) binding fit model with Biacore Insight Evaluation software v.3.0 (GE Healthcare).

### Production of vesicular stomatitis virus (VSV)-based pseudotyped viruses

The pseudoviruses of the prototype and Omicron were constructed with a replication-deficient VSV vector backbone containing the GFP gene (VSV-ΔG-GFP) and the plasmid coding sequence of the corresponding spike proteins (pCAGGS-S), as previously described^63^. HEK293T cells were transfected with 30 μg pCAGGS-S. The VSV-ΔG-GFP pseudovirus was added 24 h post-transfection. The inoculum was removed after incubating for 2 h at 37 °C. After washing the cells with PBS, the culture medium was changed to Dulbecco’s modified Eagle medium supplemented with 10% fetal bovine serum and 10 μg/mL anti-VSV-G antibody (I1-Hybridoma). The pseudoviruses were harvested 30 h post-inoculation, passed through a 0.45 μm filter (Millipore) before being aliquoted, and stored at –80 °C.

### Neutralization assay based on the pseudovirus infection

The S304 antibody was first diluted four-fold from an initial concentration of 100 μg/mL. Subsequently, each concentration was mixed with pseudoviruses of the prototype and Omicron at the same volume. The mixture was added to each well of a 96-well plate containing Vero cells in duplicates, with infected Vero cells as the positive control. After 15 h, the plates were imaged, and the number of fluorescent cells was determined using a CQ1 confocal image cytometer (Yokogawa). Statistical analyses were performed using GraphPad Prism8.

### Reporting summary

Further information on research design is available in the Nature Research Reporting Summary linked to this article.

## Supplementary information

Supplementary Information

Reporting Summary

## Data Availability

The data that support this study are available from the corresponding authors upon reasonable request. The atomic structure coordinates have been deposited in the RCSB Protein Data Bank (PDB) with the accession codes 7Y9S, 7XCP, 7Y9Z, 7XCH, 7XCI, 7YA0, 7YA1, 7XCO, 7XCK, 7YAD. The corresponding electron microscopy maps have been deposited in the Electron Microscopy Data Bank with the accession codes EMD-33690, EMD-33125, EMD-33697, EMD-33120, EMD-33121, EMD-33698, EMD-33699, EMD-33124, EMD-33123, EMD-33709. Other structures for analysis, including 6ZGG, 6LZG, 7JW0, 6VSB, 7KNB, 6WPS, 7R6X, 6WPT, 7JX3, 6VXX, 6VYB, 7BNM, 7BNN, 7BNO, 7N1U, 7N1V, 7N1Y, 7N1T, 7N1Q, 7SBS, 7SBL, 7KMZ, 7KMS, 7EDJ, 7V7Z, 7V81, 7V88, and 7V89, were obtained from the PDB. Source data are provided in this paper.

## Source data

Source Data
