# Supplementary material for: Omicron SARS-CoV-2 mutations stabilize spike up-RBD conformation and lead to a non-RBM-binding monoclonal antibody escape
Source: Nat Commun. 2022 Aug 24;13:4958. doi: 10.1038/s41467-022-32665-7 (PMC9399999; doi:10.1038/s41467-022-32665-7)
Supplement: Supplementary file 1 — Supplementary Information [file 41467_2022_32665_MOESM1_ESM.pdf]

Supplementary Information

Omicron SARS-CoV-2 mutations stabilize spike up-RBD conformation and lead to a non-RBM-binding monoclonal antibody escape

Zhennan Zhao, et al

Supplementary Figures 1-10

Supplementary Table 1

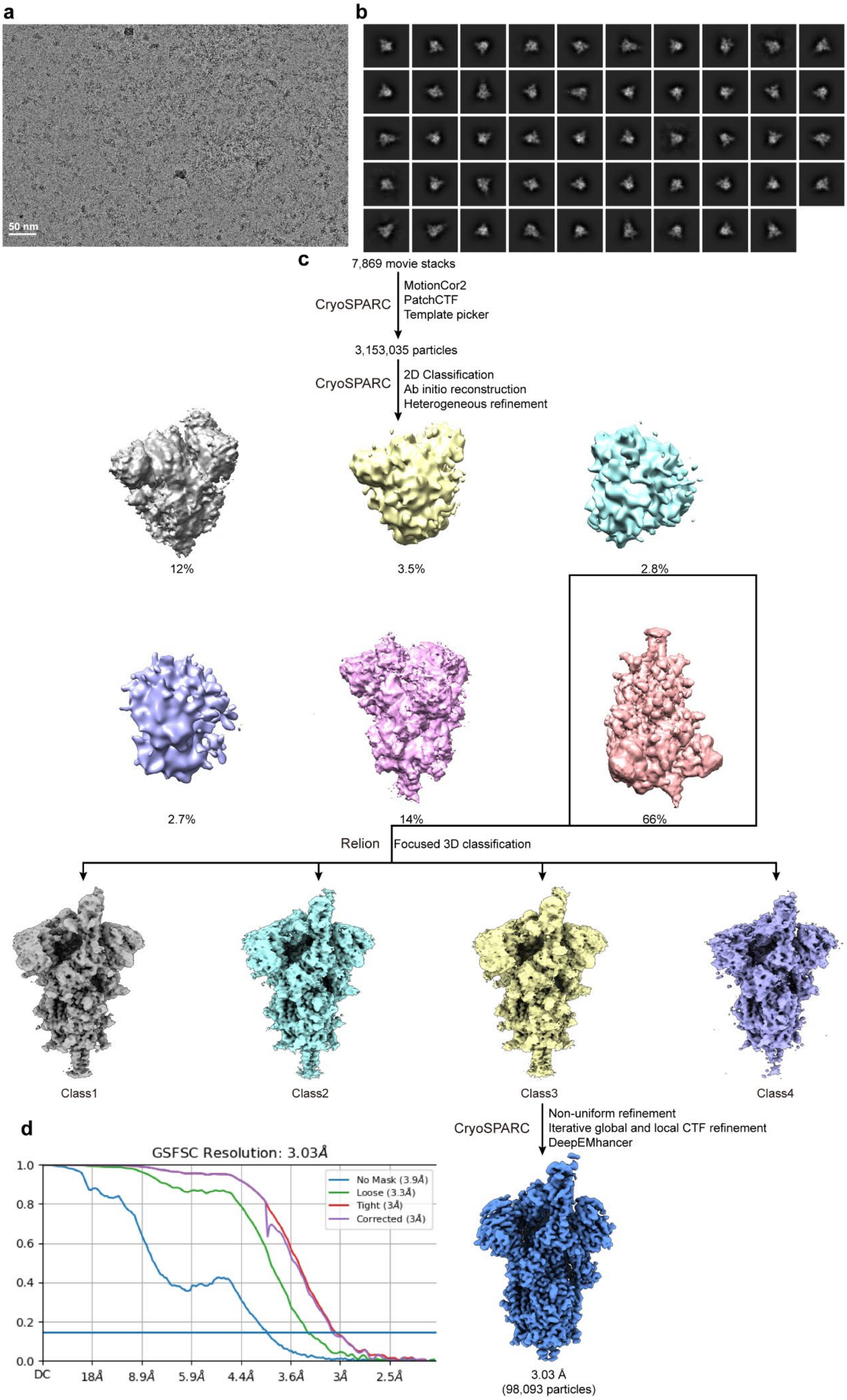

a
50 nm
b
c
7,869 movie stacks
CryoSPARC
MotionCor2
PatchCTF
Template picker
3,153,035 particles
CryoSPARC
2D Classification
Ab initio reconstruction
Heterogeneous refinement
12%
3.5%
2.8%
2.7%
14%
66%
Relion
Focused 3D classification
Class1
Class2
Class3
Class4
CryoSPARC
Non-uniform refinement
Iterative global and local CTF refinement
DeepEMhancer
3.03 Å
(98,093 particles)
d
GSFSC Resolution: 3.03Å
No Mask (3.9Å)
Loose (3.3Å)
Tight (3Å)
Corrected (3Å)
1.0
0.8
0.6
0.4
0.2
0.0
DC
18Å
8.9Å
5.9Å
4.4Å
3.6Å
3Å
2.5Å

Supplementary Fig. 1 Cryo-EM single-particle analysis of the apo Omicron spike

a. Representative one from 7,869 cryo-EM micrographs collected for the apo Omicron spike. **b**. 2D class average images of the apo Omicron spike. **c**. A brief workflow of cryo-EM image processing and reconstruction. **d**. The FSC curve for the density map at 3.03 Å resolution.

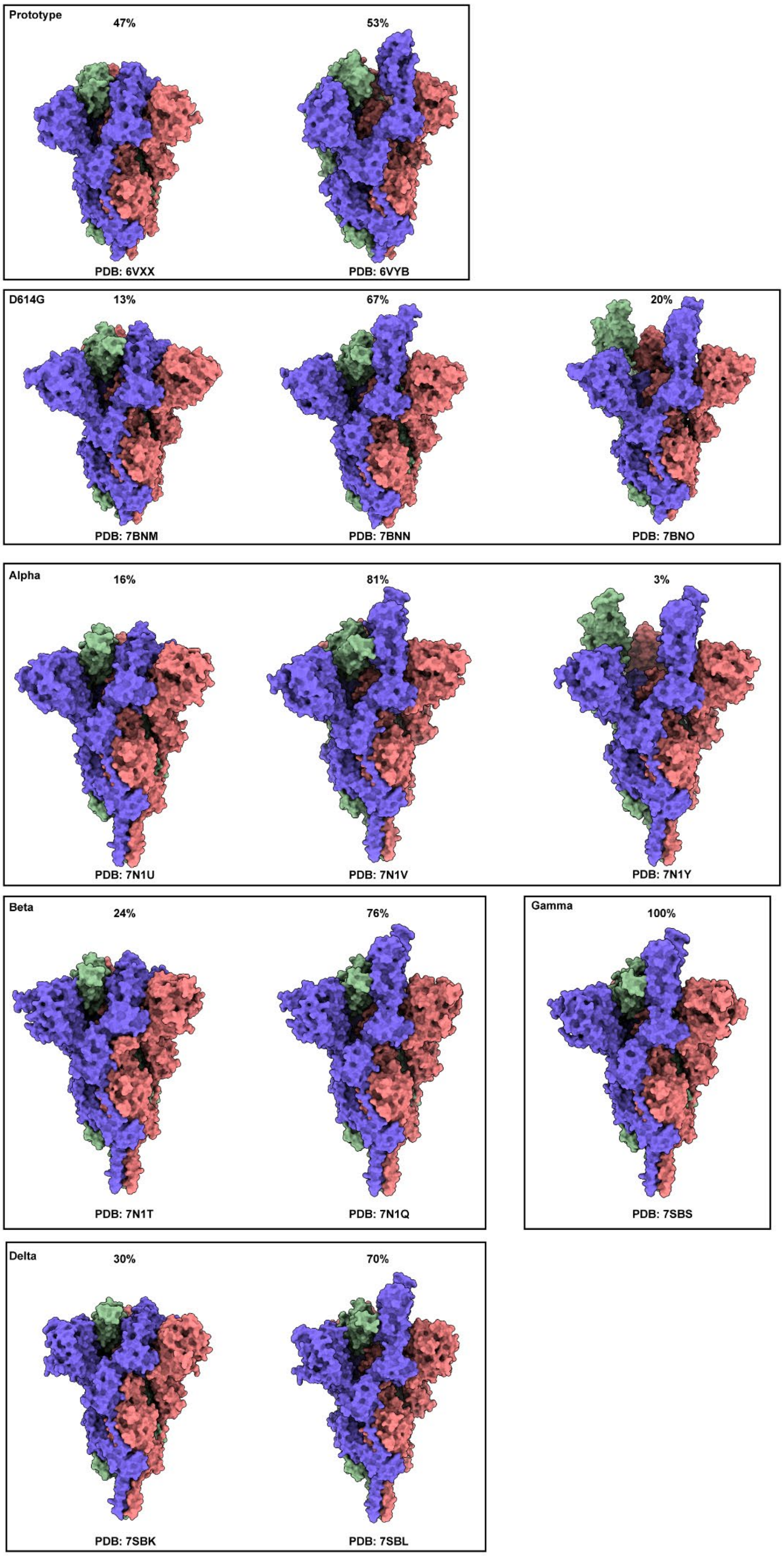
Prototype
47%
53%
PDB: 6VXX
PDB: 6VYB
D614G
13%
67%
20%
PDB: 7BNM
PDB: 7BNN
PDB: 7BNO
Alpha
16%
81%
3%
PDB: 7N1U
PDB: 7N1V
PDB: 7N1Y
Beta
24%
76%
PDB: 7N1T
PDB: 7N1Q
Gamma
100%
PDB: 7SBS
Delta
30%
70%
PDB: 7SBK
PDB: 7SBL

Supplementary Fig. 2 Conformational comparison of apo spike trimers of the prototype, D614G, and four VOCs (Alpha, Beta, Gamma and Delta)

Surface presentations of the prototype (PDB: 6VXX and 6VYB)[31], D614G (PDB: 7BNM, 7BNN and 7BNO)[34], Alpha (PDB: 7N1U, 7N1V and 7N1Y)[32], Beta (7N1T and 7N1Q)[32], Gamma (PDB: 7SBS)[33] and Delta (PDB: 7SBL)[33] spikes are shown with three protomers colored medium slate blue, light coral, and dark sea green. The ratio of each spike trimer with different conformations is shown in the figure.

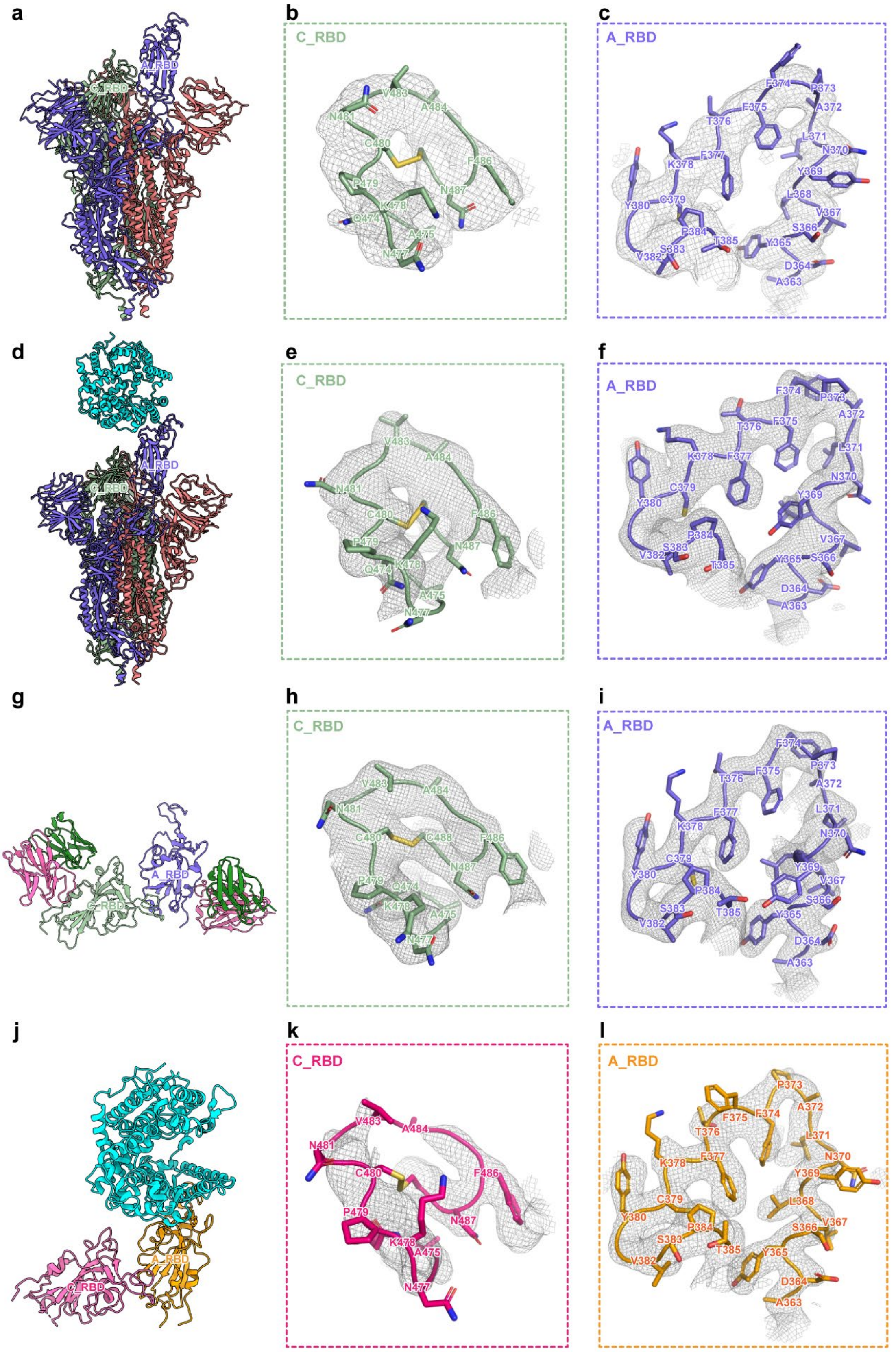

Supplementary Fig. 3 Local density maps of the interface formed by down- and up-RBDs in this study and that in the published data[27]

a-c. The local density maps of the A_RBD and C_RBD of the apo Omicron spike trimer. **d-f**. The local density maps of the A_RBD and C_RBD of the hACE2-bound Omicron spike. **g-i**. The local density maps of the A_RBD and C_RBD of the S309-RBD-RBD-S309 region which was obtained by local refinement for the S309-bound spike. **j-l**. The local density maps of the A_RBD and C_RBD of the hACE2-bound Omicron spike in the published paper[27].

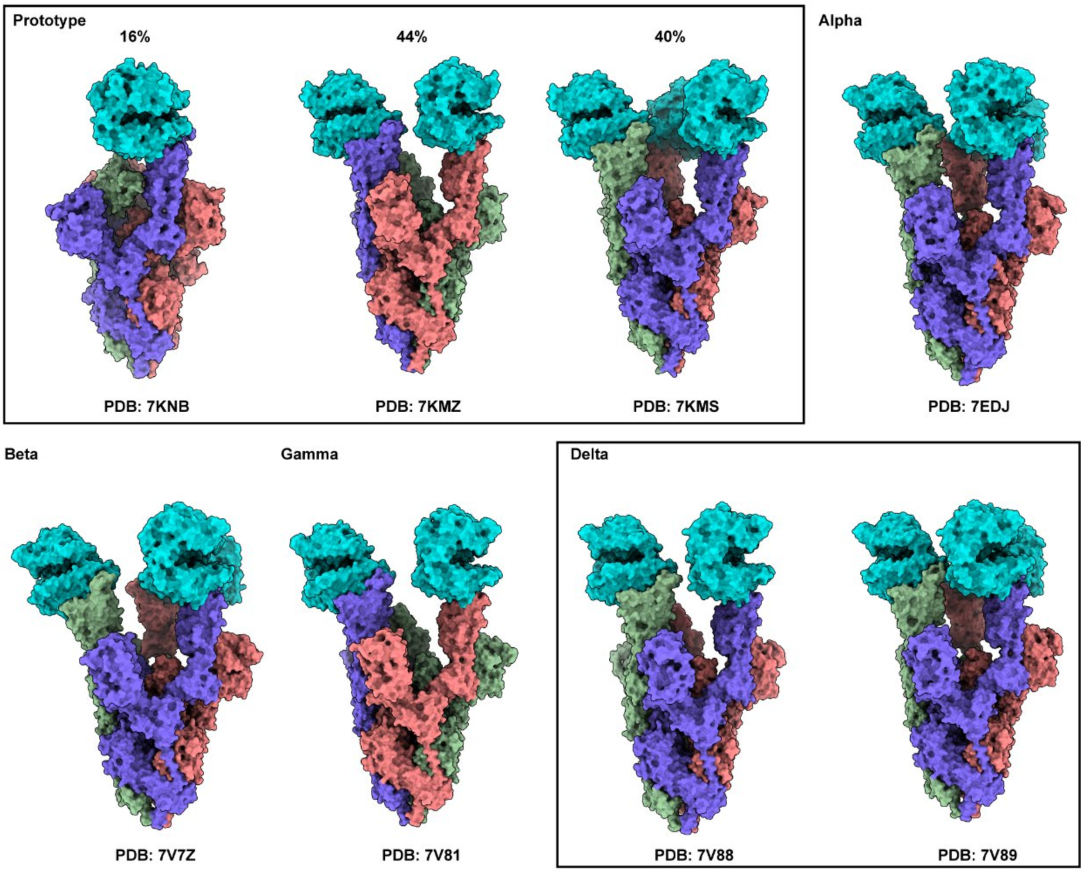

Supplementary Fig. 4 Conformational comparison of ACE2-bound spike trimers of the prototype and four VOCs (Alpha, Beta, Gamma, and Delta)

Surface presentations of the prototype (PDB: 7KNB, 7KMZ and 7KMS)[30], Alpha (PDB: 7EDJ)[35], Beta (PDB: 7V7Z), Gamma (PDB: 7V81) and Delta (PDB: 7V88 and 7V89) spike trimers in complex with the ACE2 receptor are shown with three protomers colored medium slate blue, light coral, and dark sea green, respectively. The ratios of the three conformations of the ACE2-bound prototypic spike trimer are shown in the figure.

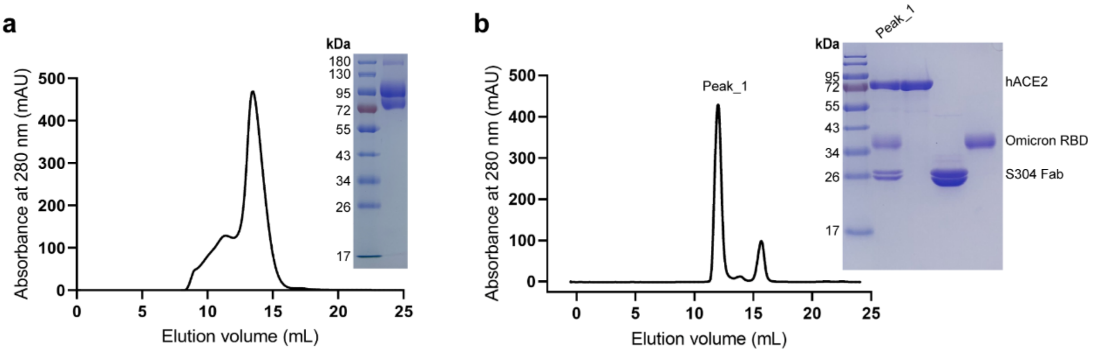

Supplementary Fig. 5 Purification of the Omicron spike ectodomain protein and the Omicron RBD-hACE2-S304 complex

a. Gel filtration chromatography of the Omicron spike ectodomain protein with HiLoad Superose® 6 increase 10/300 GL. The 280 nm absorbance curve and the sodium dodecyl sulfate-polyacrylamide gel electrophoresis (SDS-PAGE) migration profile of the pooled sample are shown. Because the furin cleavage site of this construct was not mutated, the majority of the spike was expressed and cleaved into the S1 and S2 subunits. **b**. Purification of the Omicron RBD-hACE2-S304 complex. Omicron RBD was first incubated with hACE2, followed by gel filtration chromatography of the Omicron RBD-hACE2 complex. Excessive S304 Fab was then added to the Omicron RBD-hACE2 complex and incubated overnight. Gel filtration chromatography of the Omicron RBD-hACE2-S304 with Superdex™ 200 10/300 GL. The 280 nm absorbance curve and the SDS-PAGE migration profile of the pooled sample are shown. The bands of hACE2, Omicron RBD, and S304 Fab are shown as controls. Source data are provided as a Source Data file.

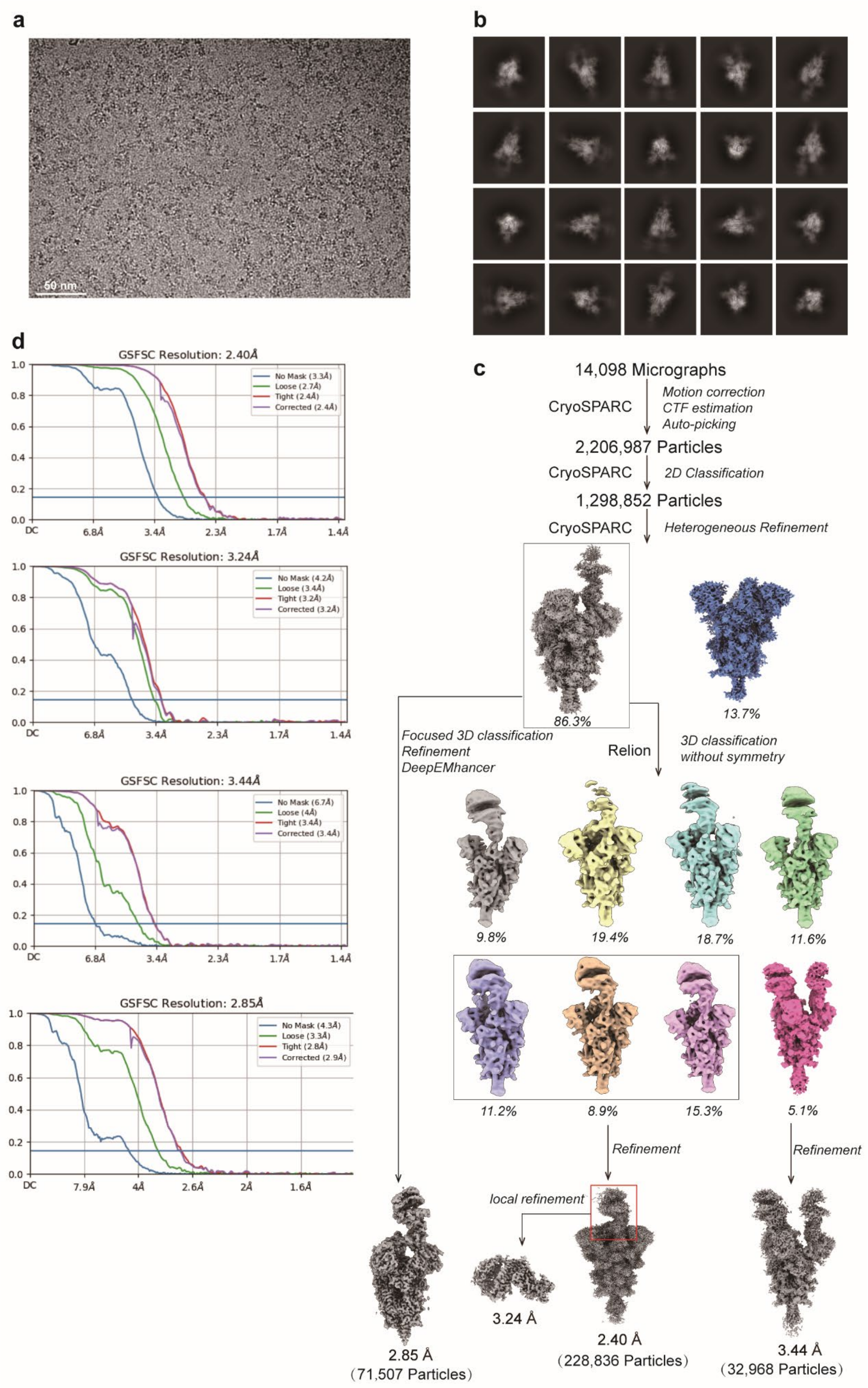

Supplementary Fig. 6 Cryo-EM single-particle analysis of the Omicron spike-

hACE2 complex

a. Representative one from 14,098 cryo-EM micrographs collected for the Omicron spike-hACE2 complex. **b**. 2D class average images of Omicron spike-hACE2 complex. **c**. A brief workflow of cryo-EM image processing and reconstruction. **d**. The FSC curves for the reconstructions.

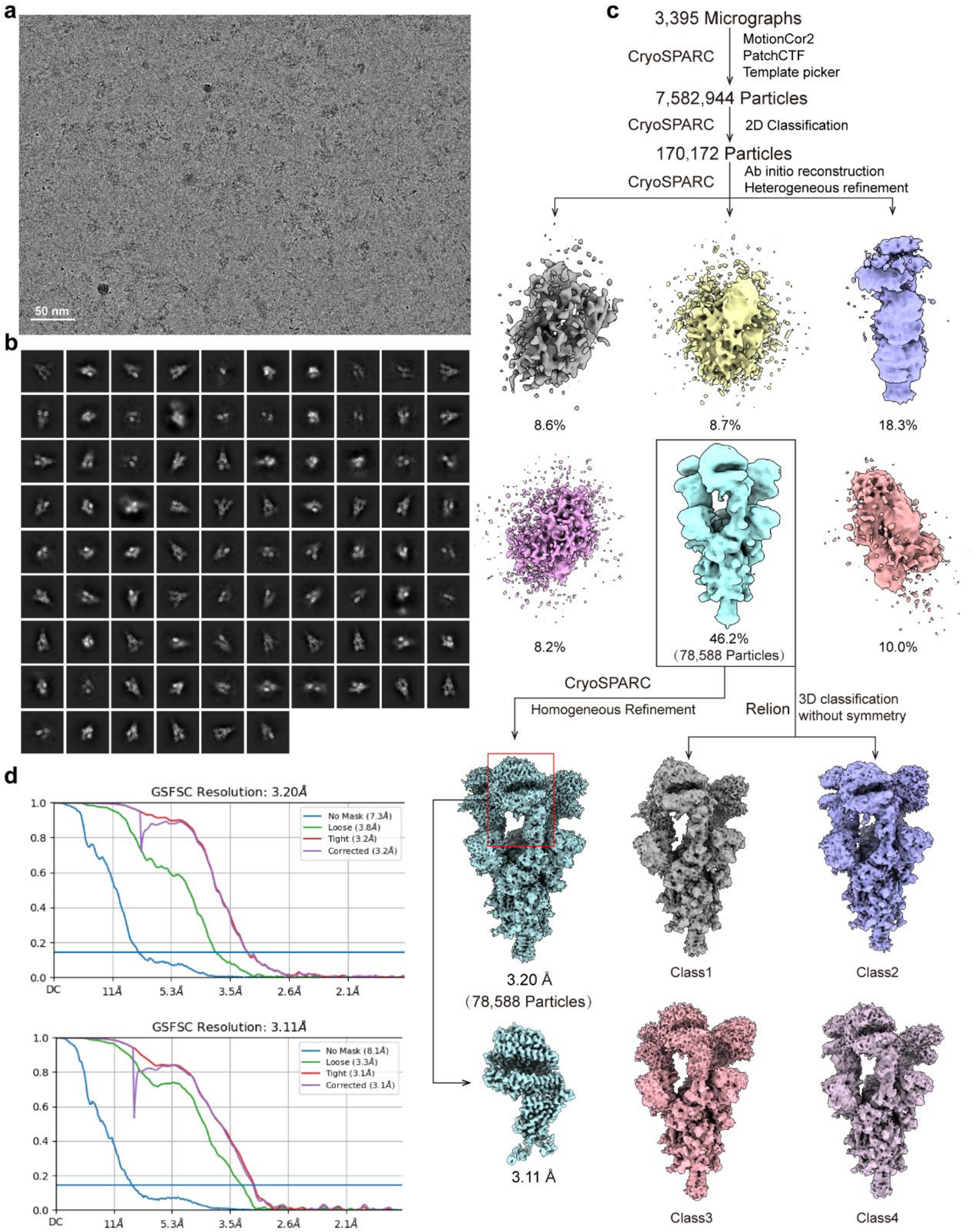

Supplementary Fig. 7 Cryo-EM single-particle analysis of the Omicron-S-L371S/P373S/F375S-hACE2 complex

a. Representative one from 3,395 cryo-EM micrographs collected for the Omicron-S-L371S/P373S/F375S mutant protein complexed with hACE2. **b**. 2D class average images of Omicron-S-L371S/P373S/F375S-hACE2 complex. **c**. A brief workflow of cryo-EM image processing and reconstruction. **d**. The FSC curves for the reconstructions.

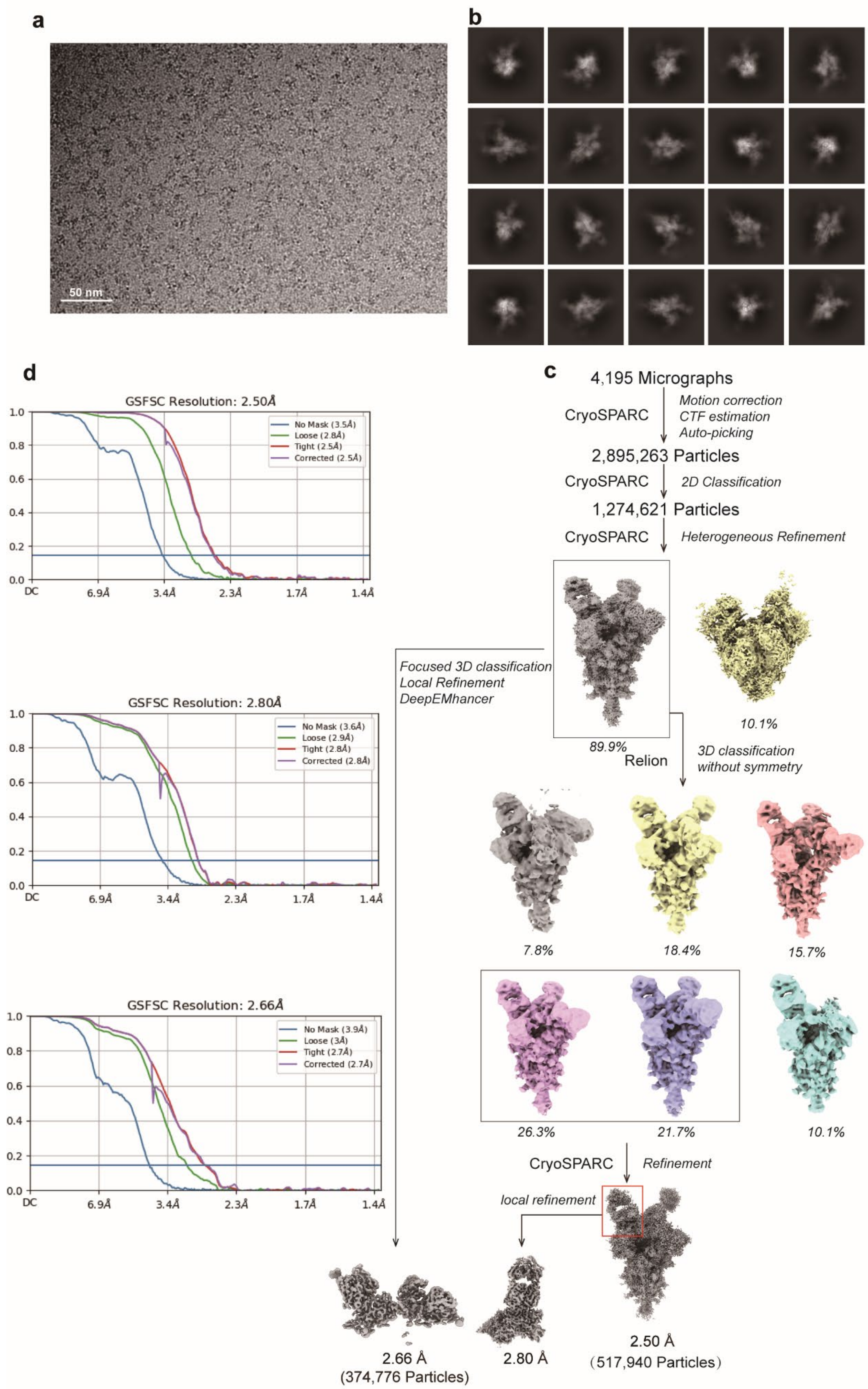

Supplementary Fig. 8 Cryo-EM single-particle analysis of the Omicron spike-S309

complex

a. Representative one from 4,195 cryo-EM micrographs collected for the Omicron spike-S309 complex. **b**. 2D class average images of Omicron spike-S309 complex. **c**. A brief workflow of cryo-EM image processing and reconstruction. **d**. The FSC curves for the reconstructions.

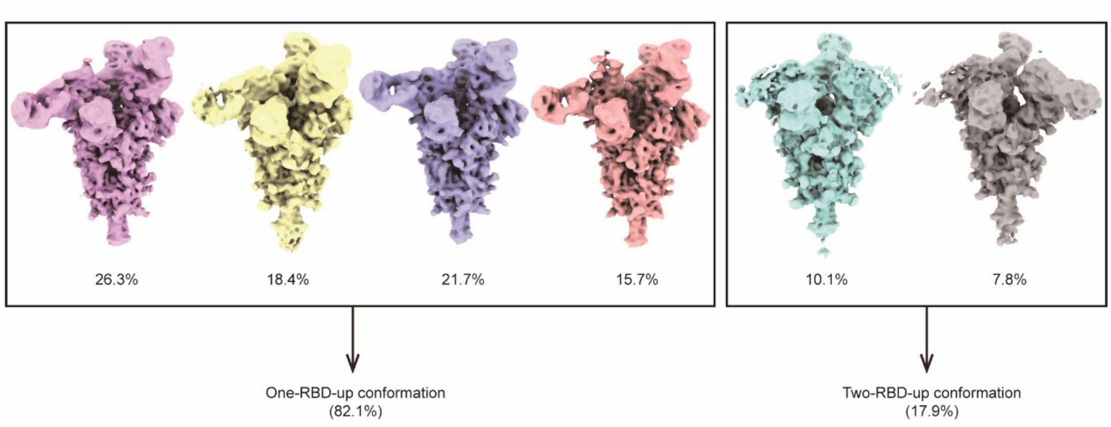

Supplementary Fig. 9 The proportions of the one or two-RBD-up conformations in the Omicron spike-S309 complex

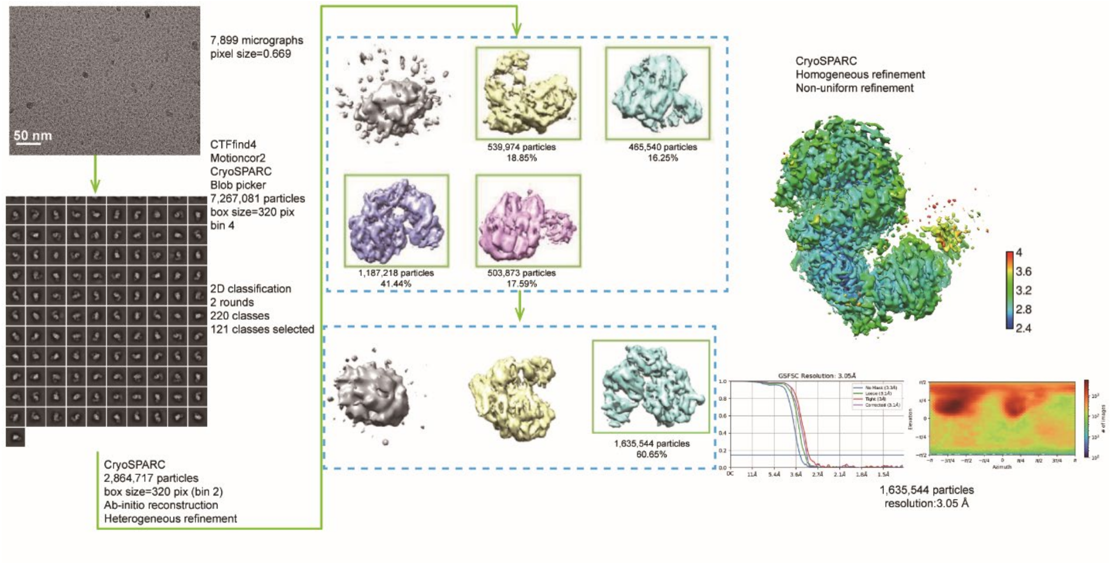

Supplementary Fig. 10 Cryo-EM single-particle analysis of the Omicron RBD-hACE2-S304 fab ternary complex

Representative one from 7,899 cryo-EM micrographs collected for the Omicron RBD-hACE2-S304 fab ternary complex. Cryo-EM images were processed and reconstructed using CryoSPARC[53].

Supplementary Table 1. Cryo-EM data collection, refinement, and validation statistics

	Apo Omicron spike trimer	Omicron RBD-hACE2-S304 Fab ternary complex
Data collection and processing		
Voltage (kV)	300	300
Electron exposure ($e^{-}/Å^{2}$)	60	50
Defocus range (μm)	-1.0 to -2.0	-1.0 to -2.0
Pixel size (Å)	1.11	0.669
Symmetry imposed	C1	C1
Final particle images (no.)	98,093	1,635,544
Map resolution (Å)	3.03	3.05
FSC threshold	0.143	0.143
Refinement		
Initial model used (PDB code)	6VSB	7R6X
Map sharpening *B* factor ($Å^{2}$)	DeepEMhancer	191.1
Model composition		
Non-hydrogen atoms	25,273	8,261
Protein residues	3,126	1,017
Ligands	53	8
R.m.s. deviations		
Bond lengths (Å)	0.004	0.002
Bond angles (°)	0.961	0.558
Validation		
MolProbity score	1.67	1.79
Clashscore	5.71	10.53
Poor rotamers (%)	0.26	0.23
Ramachandran plot		
Favored (%)	94.79	96.33
Allowed (%)	4.92	3.57
Disallowed (%)	0.29	0.10

	Omicron S-hACE2 one-RBD-up conformation	Omicron S-hACE2 two-RBD-up conformation	Omicron RBD -hACE2 (local refinement)
Data collection and processing			
Voltage (kV)	300	300	300
Electron exposure ($e^{-}/Å^{2}$)	50	50	50
Defocus range (μm)	-1.0 to -2.5	-1.0 to -2.5	-1.0 to -2.5
Pixel size (Å)	0.66	0.66	0.66
Symmetry imposed	C1	C1	C1
Final particle images (no.)	71,507	32,968	228,836
Map resolution (Å)	2.85	3.44	3.24
FSC threshold	0.143	0.143	0.143
Refinement			
Initial model used (PDB code)	7KNB	7KNB	7KNB
Map sharpening B factor ($Å^{2}$)	DeepEMhancer	150.2	130.8
Model composition			
Non-hydrogen atoms	30,837	35,749	6,547
Protein residues	3,785	4,381	791
Ligands	68	72	9
R.m.s. deviations			
Bond lengths (Å)	0.004	0.002	0.009
Bond angles (°)	0.667	0.544	0.793
Validation			
MolProbity score	1.86	1.70	1.73
Clashscore	8.32	7.63	7.82
Poor rotamers (%)	0.36	0.29	0.86
Ramachandran plot			
Favored (%)	93.80	95.93	95.68
Allowed (%)	5.83	3.86	3.81
Disallowed (%)	0.37	0.21	0.51

	hACE2-bound mutant Omicron spike trimer (L371S, P373S and F375S)	hACE2-bound mutant Omicron spike trimer (L371S, P373S and F375S) (local refinement)
Data collection and processing		
Voltage (kV)	300	300
Electron exposure ($e^{-}/Å^{2}$)	50	50
Defocus range (μm)	-1.0 to -2.0	-1.0 to -2.0
Pixel size (Å)	0.88	0.88
Symmetry imposed	C1	C1
Final particle images (no.)	78,588	78,588
Map resolution (Å)	3.20	3.11
FSC threshold	0.143	0.143
Refinement		
Initial model used (PDB code)	7KNB	7KNB
Map sharpening *B* factor ($Å^{2}$)	65.6	DeepEMhancer
Model composition		
Non-hydrogen atoms	39,996	6,525
Protein residues	4,917	791
Ligands	66	8
R.m.s. deviations		
Bond lengths (Å)	0.004	0.004
Bond angles (°)	0.966	0.738
Validation		
MolProbity score	1.87	1.78
Clashscore	9.02	6.75
Poor rotamers (%)	0.16	0.72
Ramachandran plot		
Favored (%)	94.20	93.90
Allowed (%)	5.57	5.97
Disallowed (%)	0.23	0.13

	Omicron S-S309	Omicron RBD-S309 (local refinement)	S309-RBD-RBD-S309 (local refinement)
Data collection and processing			
Voltage (kV)	300	300	300
Electron exposure (e^{-}/Å^2)	50	50	50
Defocus range (μm)	-1.0 to -2.5	-1.0 to -2.5	-1.0 to -2.5
Pixel size (Å)	0.67	0.67	0.67
Symmetry imposed	C1	C1	C1
Final particle images (no.)	517,940	517,940	374,776
Map resolution (Å)	2.50	2.80	2.66
FSC threshold	0.143	0.143	0.143
Refinement			
Initial model used (PDB code)	6WPS	6WPS	6WPS
Map sharpening *B* factor (Å^2)	86.1	102.8	DeepEMhancer
Model composition			
Non-hydrogen atoms	35,802	5,488	6,728
Protein residues	4,494	704	848
Ligands	63	3	6
R.m.s. deviations			
Bond lengths (Å)	0.003	0.004	0.005
Bond angles (°)	0.536	0.704	1.006
Validation			
MolProbity score	2.06	1.58	2.12
Clashscore	6.76	4.80	8.57
Poor rotamers (%)	2.67	0.00	2.23
Ramachandran plot			
Favored (%)	94.79	95.26	94.14
Allowed (%)	5.10	4.60	5.62
Disallowed (%)	0.11	0.14	0.24
